# On the Inhalation of Sulphuric Ether

**Published:** 1847-10

**Authors:** 


ARTICLE II.

### ON THE INHALATION OF SULPHURIC ETHER.

Some months since we gave an account of the use of ether for the prevention of pain during operations, and related some cases in which we had used it with advantage. We have since that time intended to present a summary of the observations made in different countries with the results and conclusions arrived at. But the universal approbation with which it was at first received has given place to doubt and in some cases condemnation, and the reports of cases, opinions, successes, deaths, dissections, experiments, &c., have become so voluminious and varied, as to make us despair of giving even an analysis of their contents indicating the conclusions to which they tend; for while men of science and caution are deterred from its use by some fatal results attributable to its abuse, the patients cry out for it, and the bolder, perhaps less informed of the profession, continue to use it with almost universal success.

If instead of searching among recorded cases and opinions for evidences of its value, we were to take the results of our own experience and the observations we have been able to make upon its use by others as a guide, we should not only reiterate the favorable impression formerly expressed in regard to its value in preventing pain during operations, but add also that as an anadyne it may in a great number of cases supercede opium and as a febrifuge quinine and diaphoretics. Of many cases it has never but in a single instance produced ill effects, and those of the most evanescent kind, but it has produced results so excellent that we are constrained to believe it the most admirable of discoveries.

In order, however, to make this true of its use it is necessary that care should be taken not to give it in too long or too concentrated a form, or to exclude the atmospheric air from the lungs. The method of using it which experience has shown us to be most effectual and safe is, if the receiver be employed, to allow the patient to inhale it gradually for a length of time without closing the nostrils, if after a few minutes the effect is not produced, these may be stopped. As soon as indications of its effects are observed it should be suspended and given from time to time as long as there is occasion for its use. In some instances we have employed simply a sponge held over the mouth and nostrils and when only an anadyne effect for the relief of pain without operation a small one with little ether will do.

It is absolutely essential to its safety and success that *pure rectified sulphuric ether* should be employed, and none of this is found in the shops except where care has been taken to procure it. It is for this reason that many have failed. To these directions we would add that it is not to be given to persons who are extremely timid or averse to its use as fear has an unfavorable effect, but the patient should be allowed to take it in his hands, inhale it gradually before hand, and if after such trials he is still averse to its use it should not be urged upon him. Usually after such inhalations, patients acquire confidence and find it agreeable.—Another rule is that it should never be given for an operation extremely slight, for it is unnecessary, nor in one where the patient is likely to die from its immediate effects as it might add to the danger and difficulty.

With such reservations we can safely recommend it. There are, however, states of the lungs, heart, and brain in which its use is thought not advisable.

Among these are tubercles and inflammation of the lungs and pleura. We do not, for ourselves, think these cases should altogether prohibit its use; on the contrary we suppose that it may prove useful in alleviating the dyspnea, attendant on such cases.

In valvular disease of the heart it has been thought dangerous; but we have known it to give great relief to asthmatic patients affected with such diseases.

"In persons of short necks and subject to congestion it should not be used." We think there is no doubt of the correctness of this precept.

We have lately used it for the extirpation of two cancerous breasts, extirpation of tumors, opening of abscesses, strabismus &c., with the most satisfactory results.

It has also been used in the Chicago Hospital under the direction of a pupil of that establishment, Mr. J. W. Freer, in a case of painful menstruation with the happiest result.

Also in a case of superficial burn of a most painful character, in which the patient, a young woman, was allowed a bit of sponge and a small bottle of the ether during the night, and she inhaled it uninterruptedly for six hours without bad effect. On the contrary, when it was suspended too long the pain became intense, and she cried for it "like a child for milk." But the most delightful of the results obtained by Mr. Freer have been in cases of ague. Two or three inhalations invariably arrested the paroxysm *instantaneously* during the cold stage, brought on diaphoresis, and in cases when there was no unusual exertion the paroxyms have not returned.

In labor it has been tried and various are the reports of its action. Prof. Herrick has used it once in a case of natural labor for half an hour with excellent effects. It took away the pain without in any degree interferring with the contraction of the uterus. D. B.

